# Dataplamt: base de dados bibliográfica sobre o uso tradicional das plantas brasileiras

**DOI:** 10.1590/S0104-59702026000100024

**Published:** 2026-07-24

**Authors:** Maria das Graças Lins Brandão

**Affiliations:** i Instituto Cayapiá de Defesa da Cultura e Conservação das Plantas Nativas Usadas pelo Povo Brasileiro Tiradentes MG Brasil cayapia.instituto@gmail.com Presidente, Instituto Cayapiá de Defesa da Cultura e Conservação das Plantas Nativas Usadas pelo Povo Brasileiro. Tiradentes – MG – Brasil. cayapia.instituto@gmail.com

**Keywords:** Ameríndios, Bibliografia, Biodiversidade, História natural, Plantas medicinais, Amerindians, Bibliography, Biodiversity, Natural history, Medicinal plants

## Abstract

Diferentemente das medicinas milenares como a chinesa e a *ayurveda*, registros sobre usos tradicionais das plantas brasileiras encontram-se dispersos e sem interpretação. A Dataplamt foi criada com o objetivo de recuperar e disponibilizar essas informações e já contém dados sobre 3.464 espécies, recuperadas de 114 livros, produzidos até 1950. São indicadas como medicinais 170 categorias, sendo a maior parte (28) contra doenças infectoparasitárias. Nomes originais das plantas são também recuperados, confirmando ancestralidade. A Dataplamt permite identificar usos tradicionais resilientes, importantes para o desenvolvimento de produtos fitoterápicos tradicionais. Contribui também para a bioeconomia e a divulgação junto à população das plantas da biodiversidade brasileira.

## Abstract (en)

Unlike remedies from ancient systems like Chinese traditional medicine and Ayurveda, records of the traditional uses of Brazilian plants are scattered and lack interpretation. Dataplamt was created to recover this knowledge and make it readily available, and already contains data on 3,464 species recovered from 114 texts written prior to 1950; it spans 170 medicinal categories, with 28 addressing infectious or parasitic diseases. It also includes native plant names, confirming their ancestral roots. The Dataplamt database makes it possible to identify resilient traditional uses, which are important for developing traditional herbal products, and also contributes to the bioeconomy and spreading information about Brazil’s plants to the general population.

Descobertas arqueológicas vêm confirmando que as plantas são usadas nas Américas há milênios. Pesquisas mais recentes mostram que a floresta amazônica, por exemplo, como a conhecemos hoje, foi composta de jardins bem-organizados e administrados pelos ancestrais ameríndios (Schmidt et al., 2023). Milhões de pessoas viviam ali e cultivavam centenas de plantas. Entre essas, 85 espécies hiperdominantes foram identificadas, e incluem várias palmeiras, diversos frutos, além de plantas medicinais (Steege et al., 2013). Os usos atuais dessas plantas pré-colombianas sinalizam que elas são espécies resilientes, ou seja, atravessaram séculos e chegaram aos dias de hoje, confirmando sua ancestralidade.

Durante a colonização das Américas, o uso de plantas pelos ameríndios começou a ser registrado. Um exemplo importante é a casca de espécies peruanas de *Cinchona* (quina) que produzem o antimalárico quinino. Essa planta é cultivada hoje na Ásia para obtenção dessa mesma substância visando ao preparo de medicamentos e o ingrediente amargo dos refrigerantes tônicos (Cosenza et al., 2013). Um dos mais importantes usos registrados no Brasil foi como emético e amebicida da *Carapichea ipecacuanha* (Brot.) L. Andersson (ipecacuanha). Devido a sua importância, essa espécie está incluída em várias farmacopeias do mundo, desde o século XIX (Brandão et al., 2009). Registros sobre o uso de plantas pelos ameríndios, feitos nos séculos posteriores, também são importantes e permanecem como rica fonte de informações sobre o potencial das plantas da biodiversidade brasileira (Pereira, 2024).

Desde 2014, a Organização Mundial da Saúde (OMS) passou a considerar a efetividade de uma planta medicinal também por meio de sua tradicionalidade (WHO, 2013). Essa efetividade pode ser atestada a partir de informações consistentes sobre seus usos tradicionais, por longo período, registrados em bibliografia. A OMS incentiva essa validação das plantas usadas nos sistemas médicos milenares, como a medicina chinesa, *ayurveda* e as ameríndias, em que a organização destaca ser necessário: (1) preservar o conhecimento histórico e associá-lo a dados mais recentes sobre os usos das plantas; (2) promover a criação de um banco de dados global, com informações sobre a medicina tradicional; e (3) respeitar os direitos de propriedade intelectual das pessoas que detêm esse conhecimento. A Anvisa aceita esse tipo de validação para registro e criou a linha dos produtos tradicionais fitoterápicos (Carvalho et al., 2025). Nesse contexto está a Dataplamt, base de dados histórica bibliográfica sobre usos tradicionais das plantas nativas do Brasil.

## Medicina tradicional brasileira: breve histórico

Diferentemente das medicinas chinesa e *ayurveda*, em que os usos das plantas são registrados em compêndios criados por eles próprios há milênios, informações sobre as plantas brasileiras começaram a ser registradas com a chegada dos europeus. Durante o período colonial, o Brasil permaneceu sob rígido controle português, com o objetivo de inibir o interesse dos recursos naturais por outras nações. Os jesuítas foram os primeiros a registrar informações sobre plantas utilizadas pelos ameríndios. Eles incorporaram espécies nativas aos remédios utilizados na Europa, como é o caso da *Triaga brasilica*, indicada para tratar febres e picadas de cobra (Pereira et al., 1996). A despeito dos impedimentos, naturalistas ilustres estiveram no Brasil e descreveram o uso de plantas. No século XVII, por exemplo, os holandeses invadiram o Nordeste, e, em 1648, o médico Willem Piso (1611-1678) publicou a obra *Historiae naturalis and medicae brasilis* (Piso, 1948). O alemão Alexander von Humboldt (1769-1859) também publicou informações sobre o uso de plantas pelos brasileiros no norte da América do Sul (Humboldt, Bonpland, 1808). Frei José Mariano da Conceição Vellozo (1741-1811), por sua vez, foi o pioneiro entre os cientistas brasileiros. Em 1790 ele já havia organizado uma flora com descrição botânica de 1.639 espécies, incluindo indicação de características e usos tradicionais de 153 nativas (Vellozo, 1825). Em 1808, a família real portuguesa instalou-se no Rio de Janeiro, onde permaneceu por 13 anos. A partir desse período, dezenas de cientistas e viajantes estrangeiros obtiveram permissão para entrar no país, dando início a um ciclo de expedições científicas, patrocinado por diferentes nações. Milhares de plantas foram descobertas, e seus usos tradicionais descritos em dezenas de documentos e bibliografias. O botânico francês Auguste de Saint-Hilaire (1799-1853) e o médico alemão Karl Friedrich Philipp von Martius (1794-1868), por exemplo, produziram obras específicas sobre, respectivamente, setenta e mais de seiscentas espécies, cujos usos foram observados em todas as regiões do país percorridas por eles (Saint-Hilaire, 2014; Martius, 2023).

Os remédios registrados por esses autores foram rapidamente incorporados à prática médica. O polonês Pieter Chernoviz (1812-1881) viveu por décadas no Rio de Janeiro e escreveu guias médicos amplamente utilizados na medicina convencional da época, até a publicação da primeira edição da Farmacopeia Brasileira (FBRAS), em 1929 (Ricardo et al., 2017). Espécies descritas por esses autores também integraram a primeira edição da FBRAS, entre elas, a *Anadenanthera colubrina* (Vell.) Benth. (angico) e *Ilex paraguariensis* A.St.-Hil. (chá-mate), descritas por frei Vellozo e pelo botânico francês Saint-Hilaire, respectivamente. A *Erythrina mulungu* Mart. (mulungu), *Monteverdia ilicifolia* (Mart. ex Reissek) Biral (espinheira-santa) e *Stryphnodendron adstringens* (Mart.) Coville (barbatimão), descritas por von Martius, são atualmente utilizadas para a produção de medicamentos fitoterápicos, registrados pela Anvisa.

Por outro lado, a despeito de todo o seu potencial, a vegetação nativa do Brasil vem sofrendo um contínuo processo de destruição, iniciado com a exploração do pau-brasil (*Paubrasilia echinata* [Lam.] Gagnon, H.C.Lima & G.P.Lewis) pelos portugueses. Atualmente, a destruição continua seguindo o mesmo modelo econômico de outrora, impulsionado pelo agronegócio e pela mineração. Os portugueses também priorizavam o uso de plantas exóticas e importadas em detrimento de espécies nativas. No século XVI, por exemplo, foi comemorado o sucesso no cultivo de manga, canela, gengibre e outras espécies vindas da Ásia (Ferrão, 2016).

Na década de 1950, novas políticas econômicas estimularam a industrialização, iniciando um período de profundas transformações no país (Mittermeyer et al., 2005). A urbanização ocorreu rapidamente, sobretudo na região Sudeste, transformando as cidades em centros econômicos, resultando em atração de mão de obra e êxodo rural (Alves et al., 2011). Criou-se um amplo mercado para produtos industrializados, seguido de uma sociedade de consumo, enquanto as “coisas rurais” ligadas à natureza passaram a ser classificadas como “atrasadas” (Carvalho, 2003). A invasão da indústria farmacêutica internacional, ocorrida também nessa época, por sua vez, substituiu os remédios das plantas por produtos mais “modernos”. Na segunda e terceira edições da FBRAS, publicadas em 1959 e 1977, respectivamente, o número de plantas medicinais nativas foi fortemente reduzido, dando lugar aos produtos sintéticos (Brandão et al., 2006). Infelizmente, essa tendência permanece, com a recente sétima edição (Anvisa, 2024) tendo maior número de monografias para espécies exóticas e importadas.

As consequências de todas as imposições econômicas e culturais sobre os usos das plantas medicinais nativas vêm sendo devastadoras: em várias localidades do interior de Minas Gerais, e até da Amazônia, espécies exóticas e importadas são mais conhecidas e usadas pela população, caracterizando um processo de erosão cultural (Brandão, Monte-Mór, 2008; Brandão et al., 2004). A necessidade de recuperar e disponibilizar informações sobre os usos tradicionais das plantas da biodiversidade brasileira tornou-se, portanto, importante e necessária, sendo estímulo para a criação da Dataplamt.

## A Dataplamt

Criada em 2006 pelo Centro Especializado em Plantas Aromáticas, Medicinais e Tóxicas da Universidade Federal de Minas Gerais, a Dataplamt é mantida atualmente pelo Instituto Cayapia e contém, majoritariamente, informações primárias registradas em bibliografia e documentos produzidos até a década de 1950. A base de dados não está finalizada, mas já disponibiliza milhares de informações, obtidas conforme descrito a seguir.

Seleção dos livros: as informações incluídas na Dataplamt são extraídas de obras constituídas principalmente de diários de viagens, relatórios de campo e/ou livros específicos sobre usos tradicionais das plantas. Até o momento, foram incorporadas informações extraídas de 114 livros, produzidos por 74 autores, distribuídos em dois grupos. O primeiro consiste de 26 autores que produziram suas obras de 1576 até 1807, antes da permissão de entrada de estrangeiros no país. Entre eles estão 14 portugueses, nove outros europeus e três brasileiros. O segundo grupo conta com 48 autores que produziram suas obras entre 1808 até a década de 1950. São 35 europeus de diversas nacionalidades e 13 brasileiros. As biografias dos autores estão disponíveis na página *online* da Dataplamt.

Seleção de plantas: a Dataplamt contém informações apenas sobre espécies nativas, sendo a origem de cada uma confirmada no portal *Reflora*, do Jardim Botânico do Rio de Janeiro. Cada planta da Dataplamt tem um *link* que direciona para o *Reflora*, e vice-versa, permitindo aos usuários o acesso a outras informações. Até o momento, estão incluídas 3.464 espécies com algum uso tradicional, pertencentes a 236 famílias botânicas. As famílias com maior número de espécies (mais de cem) são *Fabaceae* (505), *Poaceae* (160), *Arecaceae* (136), *Asteraceae* (111) e *Malvaceae* (102) (Programa..., s.d.).

Informações sobre os usos tradicionais: cada livro é cuidadosamente estudado, e as plantas registradas com algum tipo de uso. Plantas indicadas como medicinais vêm sendo distribuídas em 170 categorias de problemas de saúde, entre elas, 27 relacionadas a questões digestivas; 21 tegumentares; 18 do sistema nervoso; 13 do reprodutivo; dez do respiratório; oito do endócrino; sete do urinário; quatro do cardiovascular, e duas do esquelético. Outras categorias de usos medicinais são para tratar doenças infecciosas e parasitárias (28), em nutrição (24) e odontologia (4). Condições inespecíficas, como analgésico, febrífugo ou anti-inflamatório, por exemplo, também são apresentadas. Plantas citadas como úteis em reflorestamento, atração de abelhas, inseticidas ou repelentes de insetos, cosméticos, madeira, tinturaria, fibras, entre dezenas de outros usos, também estão incluídas devido à importância atual desses temas. É importante salientar que usos tradicionais em produtos comerciais devem seguir a legislação de repartição de benefícios.

Nomes originais de cada planta: todos os nomes originais das plantas descritos nas referências estudadas são incluídos, com ênfase nos ameríndios. O objetivo é contribuir para a preservação da cultura ancestral e auxiliar na destinação dos recursos advindos da comercialização de produtos industrializados elaborados com as plantas.

Local de ocorrência: os locais onde os autores descreveram observações sobre os usos das plantas também são incluídos na Dataplamt. Podem ser cidades, estados, regiões geográficas, biomas, rios, montanhas ou outros especificamente descritos pelos autores. Plantas referidas como usadas aqui e em países vizinhos (Argentina, Uruguai, Paraguai, Bolívia, Peru, Colômbia, Venezuela e Guianas) também são incluídas.

Inclusão dos dados: após a seleção das informações, os textos são digitalizados na íntegra, com a linguagem atualizada e corrigida no Word, e incluídos na Dataplamt.

## Contribuições

Recuperação e disponibilização de informações sobre usos tradicionais das plantas da biodiversidade brasileira presentes em fontes de difícil acesso: a Dataplamt contribui diretamente para o desenvolvimento de uma bioeconomia sustentável, possibilitando o acesso a tais informações estratégicas.

Direcionamento de pesquisas farmacológicas: o aquecimento global tem causado o ressurgimento de antigas doenças e o surgimento de outras, aumentando a necessidade de medicamentos inovadores. A Dataplamt disponibiliza informações sobre centenas de plantas nativas usadas para tratar doenças infecciosas e parasitárias como sífilis (178 espécies), malária (140) e gonorreia (106). As espécies com essas indicações pertencem a diferentes famílias botânicas, sugerindo um amplo leque de possibilidades para a descoberta de substâncias bioativas, com novas estruturas químicas.

Direcionamento de políticas públicas: a Dataplamt contribui na identificação de espécies resilientes, importantes para o desenvolvimento de produtos tradicionais fitoterápicos. Ricardo et al. (2018), por exemplo, desenvolveram um método no qual são atribuídos pesos aos livros sobre usos das plantas medicinais, bem como à Dataplamt. Utilizando esse método, foi possível traçar uma linha do tempo dos usos dos remédios preparados com bálsamo de copaíba (*Copaifera* spp.) e cascas de barbatimão (*S. adstringens*), desde os primeiros registros feitos pelos portugueses. Ficou evidenciado que, apesar de atualmente recomendado para diferentes fins, o uso dessas plantas como cicatrizante de feridas é o resiliente. Eu defendo a ideia de que plantas com usos resilientes deveriam ser consideradas prioritárias em programas de conservação, cultivos agroecológicos, implementação de sistemas agroflorestais e no desenvolvimento de produtos para a bioeconomia (Palhares et al., 2021).

Instrução da população brasileira sobre a importância das plantas da biodiversidade: desde 2004, nosso grupo vem também desenvolvendo uma série de ações em escolas públicas do interior do Brasil, mostrando a importância da sociobiodiversidade brasileira. Um produto recente é a coleção de livros didáticos “Velosinho e Joaquim e as plantas medicinais brasileiras”. Ela traz histórias em quadrinhos nas quais o botânico brasileiro frei Vellozo busca, atualmente, as plantas nativas citadas por ele em sua obra (Publicações, s.d.). Jogos e atividades lúdicas incluídas em cada volume complementam o aprendizado. É necessário promover tais atividades de divulgação científica em todas as regiões do Brasil, e a Dataplamt traz inúmeros exemplos de personagens e plantas para isso.

## Perspectivas

Mesmo estando incompleto, o Dataplamt já reúne informações estratégicas que contribuem para a valorização da cultura e do uso sustentável das plantas nativas do Brasil. Os próximos passos serão: (1) completar a inclusão dos dados presentes em uma centena de novas fontes; (2) criar um *link* específico para a busca por autores; (3) elencar e divulgar as espécies resilientes, especialmente as que têm uso pré-colombiano; e (4) buscar vínculos com outras bases de dados que complementem as informações.

## Data Availability

Não estão em repositório.
